# Cognitive impairment and depression in heart failure: ‘cardiological giants’

**DOI:** 10.1007/s12471-021-01595-2

**Published:** 2021-06-17

**Authors:** D. Pons, R. W. M. M. Jansen, M. E. W. Hemels

**Affiliations:** 1 Department of Geriatric Medicine, Northwest Clinics, Alkmaar, The Netherlands; 2 Department of Cardiology, Rijnstate Hospital, Arnhem, The Netherlands; 3 Department of Cardiology, Radboud University Medical Centre, Nijmegen, The Netherlands

In elderly patients with heart failure, the prevalence of non-cardiovascular comorbidity is very high and exceeds that of cardiovascular conditions [1]. Screening for ‘geriatric giants’, especially depression and cognitive impairment, in patients with heart failure is highly relevant with regard to heart failure prognosis, quality of life and medical costs due to exacerbations and hospital admissions [2, 3].

In this issue of the *Netherlands Heart Journal*, Oud and coworkers aimed to determine if cognitive impairment and depressive symptoms were recognised by their clinicians in an outpatient population of elderly heart failure patients (*n* = 157, median age 79 years) [4]. The majority had New York Heart Association (NYHA) class 2. The prevalence of cognitive impairment, defined as a Montreal Cognitive Assessment test (MoCA) score of 22 or lower, was 36%. The prevalence of depressive symptoms, defined as a Geriatric Depression Scale (GDS) score higher than 5, was 13%. The result of the primary endpoint is impressive; cognitive impairment was not recognised in 48% of the cases and depressive symptoms in 52%. It becomes painfully clear that clinical judgement of these highly prevalent geriatric comorbidities is insufficient. The authors performed excellent work, for the first time quantifying missed diagnoses, which reveals the size of the problem.

The authors clearly explain the vicious circle in which these comorbidities worsen heart failure and vice versa. They also make a fair point that detecting them is very relevant, since they strongly increase hospital admissions, mortality and disability, in part because of insufficient heart-failure-related self-care and medication adherence [5]. Cognitive impairment and depressive symptoms also lead to high costs and low quality of life [3]. Even if there were no therapy for depression or cognitive impairment, diagnosing these syndromes would be beneficial for organising the right care and thereby disrupting the downward spiral. Another strong argument in favour of screening and diagnosing these syndromes is the possibility that improving the treatment of heart failure may reduce the incidence of depressive and cognitive symptoms [3].

The prevalence of depressive symptoms in the present study may seem low compared to that in other studies [6], which can be explained by the outpatient setting and a population with relatively mild heart failure (87% NYHA class I/II). The prevalence of cognitive impairment is, however, a major eye opener. Instead of following the same trend, the prevalence of cognitive impairment may seem comparable to that in other studies [7], but is actually unexpectedly high when considering the 3 points lower MoCA cutoff value, the low NYHA class and the outpatient setting.

Although the exact value was chosen arbitrarily, we agree with the lower MoCA cutoff value for several reasons. First, the original validation study was performed in a relatively healthy cohort. Later research in a large population-based ‘cardiovascular’ cohort has produced normative data for the MoCA [8]. It became clear that MoCA scores are not only highly dependent on age and educational level, but also generally lower in patients with cardiovascular disease. Second, several studies have already shown that MoCA values < 24 are associated with worse consulting behaviour [9, 10].

The second aim of the study was to verify the impact of depressive symptoms and cognitive impairment on negative outcomes. In this study no association was found with mortality and hospital admissions. As mentioned by the authors this is probably related to the small cohort and the short follow-up period. In addition, the low NYHA functional class explains the relatively low prevalence of depressive and cognitive symptoms, further reducing the number of their resulting complications. However, earlier research has well established the impact of cognition and depression on these endpoints [3].

Screening geriatric patients with heart failure is difficult, since there are usually many instruments and little comparative evidence. In this study a step-up strategy was chosen to detect depressive symptoms, starting with two well-known questions derived from the core criteria (Diagnostic and Statistical Manual of Mental Disorders, DSM), in this article referred to as the GDS‑2. This is a very elegant way to screen for depression in heart failure patients (short and reliable), as these questions are the two core criteria for diagnosing major depression (DSM). As a positive answer to one of these questions is obligatory for the diagnosis, it is by definition unlikely that a case of depression will be missed. Conversely, to prevent overdiagnosis, an additional instrument is used to gain higher specificity, such as the Patient Health Questionnaire (PHQ-9) [11] or GDS-15. In heart failure patients the PHQ‑2 and PHQ‑9 have been most widely used, since American guidelines recommend these instruments in cardiac (especially coronary heart disease) patients. However, there is no comparative evidence in heart failure patients and the GDS-15 is an extensively validated instrument in the elderly.

Although European guidelines consider screening for depressive symptoms good practice, they do not yet recommend screening for cognitive impairment [12]. Increasing evidence in the last 15 years indicates the MoCA to be the most suitable instrument for patients with heart failure because of its ability (in contrast to the Mini-Mental State Examination questionnaire) to detect (mild) impairments frequently seen in patients with heart failure, such as executive dysfunction [13]. However, although it is widely used, it is time-consuming and there is little experience in cardiology clinics. The striking results of this study, in which relevant cognitive impairments are nearly 3 times as frequent as depressive symptoms, and missed by clinicians in half of the cases, would mean that we should especially focus on cognitive screening.

On behalf of the Netherlands Society of Cardiology (NVVC), we are currently developing new standards for organising heart failure care. If the MoCA is not appropriate for a heart failure outpatient clinic, we aim to achieve better awareness. To gain a first impression of cognitive functioning is not difficult; it just takes a few simple questions. For example: Does the patient know why he visits the doctor? Did he remember to attend the appointment himself? Can he name some of his important heart failure medications? Can he remember recent events, such as news headlines or what he had for dinner yesterday? In conclusion, early recognition of these ‘cardiological giants’ is of clinical importance.
